# The social impact of distance learning in Roman schools: “Success,” social innovation, teaching practices

**DOI:** 10.3389/fsoc.2023.1141435

**Published:** 2023-04-05

**Authors:** Veronica Lo Presti

**Affiliations:** Department of Communication and Social Research, Sapienza University of Rome, Rome, Italy

**Keywords:** distance learning, social impact, Positive Thinking Evaluation, digital skills, didactic innovation

## Abstract

Faced with the national emergency linked to the spread of COVID-19 in Italy, digital technologies have made it possible to carry out the ordinary activities of the various educational agencies through the main tool of Distance Learning (DaD). The intensive use of information and communication technologies (ICT) and the guarantee of accessibility represent an enabling prerogative for current education systems, enriching training in a variety of ways and opportunities which must be accessible to all. ICTs take on a propulsive function for change in schools because they make it possible to affect the school setting, to transform the learning environment by redesigning space, reorganizing time, modifying communication and socialization processes, encouraging, in students, the development of key competencies in digital literacy and media education. Considering the context of a school transformed and renewed by the teaching and training potential of ICT, it becomes central to reconstruct the requests and needs developed by the practitioners of educational policies to cope with the reorganization of teaching methods and times at the time of DaD. Starting from these premises, the paper focuses attention on the social impact (Stern, 2016) of DaD to evaluate: the extent and intensity of the methodological-didactic innovation required of teachers for the organization and conducting remote lessons; the increase—in students—of transversal and digital literacy skills (team working, problem solving, etc.) potentially associated with the use of ICTs; the involvement and collaboration of families in the process of assessing and verifying learning. The reflection is part of a broader research project by the University of Sapienza University of Rome entitled “The social impact assessment of DaD after COVID-19”; a 3 year evaluation research addressed to a typological sample of upper secondary schools in Rome classified on the basis of the Infrastructure and Equipment indicator of the Rav and the social effect of the school (school effect) on the academic performance of students in the tests Invalsi. The evaluation aimed to identify—from the DaD experience—indications useful in re-designing the school's intervention strategy in the phases following the pandemic; for this reason it adopted an analysis perspective that valorized the positive and most successful aspects in the testimonies of the teachers and students involved in the first phase of the research (conducted in May–June 2021). Within the framework of the Positive Thinking Evaluation, the empirical evidence—collected through the administration/conduction of semi-structured interviews, focus groups, online ethnographic observation of the lessons in DaD—will allow us to reflect on some dimensions of success and of particular social innovation for the teachers' teaching practices and the students' learning processes in DaD. In the Positive Thinking Evaluation, success is a positive effect (not just a “good practice”), even an unexpected one, of an activity that has produced a positive change in the context of program implementation.

## Introduction

This paper focuses attention on the social impact (Stern, 2016) of DaD (Distance Learning during COVID-19 pandemic) in order to assess: the extent and intensity of the methodological-didactic innovation required of teachers for the organization and delivery of distance learning lessons; the growth—in students—of transversal and digital literacy skills (team working, problem solving, etc.) potentially associated with the use of ICT; the involvement and collaboration of families in the process of assessing and verifying learning. The reflection returns the first results of the background research conducted as part of the University project (Sapienza, University of Rome) on “The social impact assessment of DaD after COVID-19”; addressed to a typological sample of schools in Rome with students aged 14–19. The evaluation aimed to identify—from the DaD experience—indications useful in re-designing the school's intervention strategy in the phases following the pandemic; for this reason it adopted an analysis perspective that valorized the positive and most successful aspects (Stame and Lo Presti, 2015; Lo Presti, 2019) in the testimonies of the teachers and students involved in the first phase of the research (conducted in May–June 2021).

Within the framework of the Positive Thinking Evaluation (Lo Presti, 2020), the empirical evidence—gathered through the administration/conduction of semi-structured interviews, focus groups, online ethnographic observation of the lessons in DaD—made it possible to reflect on some dimensions of success and of particular social innovation for the teachers' teaching practices and the students' learning processes in DaD. It is useful to point out that Distance Learning is not an entirely new practice in Italian educational contexts.

Other studies have focused their attention on the dominant role that falls to teachers as practitioners of educational policies and technological-didactic environments (Grimaldi, 2006) in order to transform the didactic and training potential of the Internet into good net-education practices—the latter defined as the set of didactic activities and learning processes that make massive use of the Internet as a technological infrastructure, and the web as a cultural repository. The interest regarding these policies and environments relies in their aim toward the needs of a generation of students—the digital natives—born within the context of the change taking place, accustomed to socializing with the school environment through digital technologies (Prensky, 2001; Ferri, 2011). Central to this research is the capacity of the new learning environments to stimulate student interaction, enhancing the cognitive and also the emotional component of learning, in order to guarantee the concrete development of transversal competences (social, citizenship, learning to learn) and of digital literacy—which appear increasingly central within the school curriculum.

## Materials and methods

### Evaluative thinking and social impact of digital in schools

Awareness of the multi-dimensional and changing nature of an educational program such as the DaD required the construction of an evaluation plan that took into account the elements of complexity and uncertainty, linked to the changes that educational policies have undergone and may undergo depending on the progress of the pandemic and the connection with other economic and social policies, which are having—and are expected to continue to have—a very significant impact on the life-worlds of students and their families.

The OECD DAC Network on Development Evaluation (EvalNet) has defined six evaluation criteria—relevance, coherence, effectiveness, efficiency, impact and sustainability—and two principles for their use. In this perspective, social impact evaluation means the qualitative and quantitative evaluation, in the short, medium and long term, of the effects of the activities carried out on the reference community with respect to the identified objective (Stern, 2016).

These criteria provide a normative framework used to determine the merit or worth of an intervention (policy, strategy, program, project or activity).

The adoption of evaluative thinking (Stame, 2007; Van der Knaap, 2017; Vo and Archibald, 2018) if useful for trying to capitalize on what is being experienced and learned from the DaD experience. From this crisis situation the adoption of a positive thinking approach—open to reconstructing the most successful stories (Tendler, 1992; Lo Presti, 2020) found in the testimonies of the teachers, students and families involved in the observation processes—has been able to offer, right from this first phase of background research, a relevant contribution to the understanding and critical analysis of complex programs (e.g., distance learning). The reconstruction of the actions and operational strategies implemented by the teachers allowed i. the functioning of distance education; ii. the improvement of student interaction and understanding in the new digital learning environments; iii. the reconciliation of the vital worlds that revolve around the student (school and family). In order to take a snapshot of the initial effects of DaD and DDI (blended or hybrid learning during the COVID-19 pandemic) on schools, students and families, an initial research phase was planned to reflect on how the interactions between teachers, families and students involved in the new DaD learning environments have evolved/are evolving.

In the digital inequality framework (Büchi and Hargittai, 2022; Decataldo and Fiore, 2022), it's very important to transform the didactic and training potential of the Internet into good *net-education* practices—that defined as the set of didactic activities and learning processes that massively use the Internet as a technological infrastructure, and the Web as a cultural source—other studies have focused their attention on the dominant role played by teachers, as practictioners of educational policies and technological-didactic environments (Grimaldi, 2006; Gui and Büchi, 2019) aimed at the needs of a generation of students—the digital natives—born within the context of change in act, used to socialize with the school environment through digital technologies (Prensky, 2001; Ferri, 2011). Central to these researches is the ability of new learning environments to stimulate student interaction, enhancing the cognitive and at the same time emotional component of learning (Buckingham, 2003), in order to guarantee the concrete development of transversal skills (social, citizenship, learning to learn) and digital literacy—which appear increasingly central to the school curriculum (Büchi, 2021).

Starting from this theoretical framework, the background research conducted in February–June 2021 involved seven schools in Rome with students aged 14–19, classified on the basis of a typological indicator that took into account: (a) the level (high, medium, low) of infrastructural and technological endowment of the schools (RAV, 2019–2021) and (b) the school effect (positive or negative) on the scholastic performance of 2nd grade students on the Invalsi tests in the basic disciplines (Italian and mathematics; RAV, 2019–2021/Source INVALSI).

In the inter-temporal comparing we have: (i) exploratory research phase (full emergence from COVID-19); (ii). extensive research phase (exit from emergence).

The exploratory research involved seven upper secondary schools in Rome and included an articulated research design, with the carrying out of focused interviews with class teacher coordinators; netnographic observation of lesson hours carried out in distance/blended learning (DL/BL) mode and focus groups with the students of classes that had gone back to presence in the 2020/2021 school years.

In terms of the activities carried out, the details are as follows:

(i) Twenty-six interviews with teachers; (ii) Seventeen hours of observation (1 h for each class); and (iii) Three focus groups.

The information collected through the interviews, focus groups and netnographic notes was organized through the use of a *meta-data sheet* that allowed for an indepth study of the experiences of the teachers and students with reference to the changes that occurred (in DL/BL times) on: (i) teachers' educational practices; (ii) students' learning modes; and (iii) teacher-student and student-student relations.

The indicators used for the ranking of schools reflect the importance of valuing infrastructural and contextual factors—such as the availability of a good internet connection and the social and cultural background of the students—, on which the main effects of digital didactics in terms of their effects on the strengthening/weakening of learning processes are assumed to depend.

The hook-up activities for recruiting schools took place during the months of March–June 2021; with the School contact persons of the reached schools it was agreed to involve two sections (one with higher students' academic performances and one with lower ones) from which two classes would be drawn to be included in the analysis and observation activities. More closely, 26 semi-structured interviews were carried out with the coordinating teachers of the selected classes, 17 h of online ethnographic observation (1 h for each class) of the lessons held in DaD/DDI mode and three focus groups with the classes returned to 100% attendance after the school decree adopted in May.

During the interviews, particular attention was paid to the reconstruction of the didactic-educational practices developed by the teachers in order to trace the first effects of DaD/DDI:

- On the technological and methodological-educational innovation of school communities;
- On the level of student learning in terms of increasing transversal skills and digital literacy;
- On the strengthening of the “teacher–student” “student–student” relationship and “school–family” communication.

## Results

### The social effects of DaD on teaching practices

The background investigation made it possible to understand the extent to which the starting conditions of the schools, in terms of technological and infrastructural endowment, represented diriment variables for reconstructing the first effects of DaD/DDI on the didactic/educational practices followed by the teachers—including in the analysis the germination of new geometries of school-family and student collaboration.

The information collected through the interviews, focus groups and netnographic notes was organized through the use of a meta-data sheet that allowed for an in-depth study of the experiences of the teachers and students with reference to the changes that occurred (in DL/BL times) on: (i) teachers' educational practices; (ii) students' learning modes; and (iii) teacher-student and student-student relations.

The three profiles of didactics that emerged from the analysis are articulated, and differ, in relation to: (i) organizational aspects concerning the methods of didactic delivery; (ii) the construction of the didactic pathway, highlighting the different declinations of learning assessment in the new digital learning environments; and (iii) relational aspects, highlighting the ambivalent responses of students to digital didactics.

The thematic analysis made it possible to reconstruct three teaching configurations: *innovative, traditional*, and *depowered*.

#### Innovative didactics

The contexts included in this first configuration present the following characteristics: (i) virtuous classes (higher academic performances), which belong to experimental sections; (ii) inserted in Istituti di Istruzione Superiore (I.I.S.) with both low and high technological endowment, located in central and more peripheral municipalities of Rome—but with both an average and positive school effect on students' scholastic performance. The effectiveness of participation in digital didactics at the time of COVID-19 is presumed to depend on social dynamics and family background. Although each school/class context is internally heterogeneous from this perspective, within this first configuration some structural factors appear to be diriment for an analysis that wishes to reconstruct mechanisms (Pawson and Tilley, 1997) and indicators of change linked to the interaction between the characteristics of the didactic offer and the needs of teachers, students and their families. In detail, the classes involved in the observation/focus group processes reproduced virtuous examples of teaching strategies that, even in pre-COVID times, stood out for: (i) an integrated use of traditional and laboratory teaching; (ii) an intensive use—by both teachers and students—of digital applications; (iii) an assiduous involvement of students in project-based learning; and (iv) an openness to peer-to-peer assessment. On the other hand, on the infrastructural front, the acceleration of the school's digitization would seem to have been facilitated by the multiple funding provided by ministerial decrees, issued during the COVID-19 period, together with other resources from the National Digital School Plan (PNSD). Focusing on the characteristics of the settings, the internal coherence of this first profile of didactics appears evident, considering the strength of the association between the innovation of teaching practices and immediate receptivity of learning. A diriment dimension that undoubtedly contributes to the characterization of this profile is represented by the intensive use of innovative teaching methodologies, to which is associated an attentive, active and proactive participation on the part of the students. With reference to the process of assessing and verifying learning, teachers' reflections on the use of innovative teaching methodologies, such as those that resort to peer-to-peer assessment that empower and engage the student more on the learning process than on performance, focus on the meanings of distance assessment. Under emergency conditions, the rethinking of the teaching strategy implied a revision of the learning assessment criteria, together with a shift of focus from performance to process, with a view to a profound reworking and enhancement of the teacher/student relationship.

The functionalities of the digital applications (optical reader, speaker) and the new features of the teaching offerings (availability of audio files, videos, etc.) have increased the involvement of students with BES (Special Educational Needs) certifications to whom, in times of “normality,” have not always been offered the possibility of benefiting from the preparatory functionalities of digital teaching.

From the characteristics drawn from this first configuration of DaD/DDI, conditions that do not always facilitate the development of sociality and interaction emerge sharply. Digital didactics, when enabled by favorable material conditions (e.g., adequacy of connection and home space), has brought about considerable changes, sometimes upheavals, in the way relationships within the classroom and within the family are experienced:

“Very often, during questioning, my parents would stop and listen, and I would be scolded if I did not get the grade they expected of me… This situation, although I maintained a high average, made my life more difficult.” (FG_03)

The student's testimony denotes an interference of the private sphere in the school sphere, which is also reflected in the families' definitions of their approach: motivating but uncooperative during the learning assessment; purposeful but interfering in matters pertaining to the teacher; demanding about profit but justifying their children's shortcomings and low performance. These are the pairs of ambivalent adjectives that recur most often in the teachers' words. On the one hand, if these adjectives refer to the positive changes that digital communication would have imprinted on the school/family relationship (in terms of immediacy and the sought-after involvement of parents), on the other hand, they highlight the problematic effects of parental interference on the evaluation of students' performance, such as complaints about the teaching load and contestation of grades (aspects that make the teacher/student relationship more complicated). Nevertheless, in spite of the difficulties, in conditions of innovation of the educational offer, the students' approach to digital didactics appears aware (of the merits and difficulties of the situation) and proactive (with respect to the ways of using digital opportunities). In reactive/relational students, the use of distance learning can open up various opportunities, including that of re-appropriating their free time, mixing study and passions. The need for relationality, which emerges in the contrasting definitions of the DaD experience (FG_01), can be articulated in responses that are as creative—such as the organization of online communities of students through meeting spaces in which it becomes important to value being together in emergency conditions—as aware of the difficulties encountered, in terms of performance and relationships, in the most vulnerable students.

#### Traditional teaching

The second configuration includes virtuous and non-virtuous sections/classes of the selected schools. The analytical criterion, adopted to trace recurrences and inter-sectionality in the reconstruction of the first social effects of DaD, is once again represented by the didactic-educational practices of the teachers that, in a manner distinct from the profile outlined above, provided/supplied the didactics with a traditional type of approach—which seems to work best especially in the mathematics and physics hours. Within this configuration, DaD/DDI appears to be an adaptive type of response to COVID-19 pandemic: the (remote) arrangement of the classic traditional lesson can have both positive repercussions (on the more motivated students, especially in the 2nd and 3rd year classes), and negative ones (at the relational level: horizontal interaction between students would appear to be penalized, while the only possible interaction would be vertical, between teacher and student).

In terms of teaching practices, the organization of group work took place without the preparation of innovative teaching methodologies; the use of materials to support the lesson (such as power points, videos and audio-recorded lessons) on the one hand allowed the most willing and motivated students to better understand teachers' explanations, on the other hand it represented an obstacle to the involvement and participation of the “less talented.” The DaD/DDI experience can then be considered a “sounding board” of social inequalities, which has amplified and sharpened cultural and social differences between students who are more or less equipped with those tools that are indispensable for interacting with the new way of doing school (appropriate devices, stable Internet connection, capacious home spaces).

Students' participation and effective performance in lessons and in group work depended indeed on both the adequacy of material resources and the presence of motivational ones. The difficulties in managing a distance lesson (not only infrastructural but also relational) made the process of assessing and verifying learning more complicated. A critical aspect of assessment—directly connected to the physical distance from the student—is linked to the performance of learning tests, which are very often distorted by the dynamics of collaboration and copying. For this reason, teachers have remodeled the format of the tests, organizing tests to be carried out within a pre-established timeframe, or preferring assessment by oral tests (taking the written ones at school, during DDI periods). In the teachers' testimonies, the conjunctural aspect of digital didactics can be fully grasped considering that, when delivered in conditions unfavorable to learning, it would seem to amplify the gap between students that are more or less motivated to study, and students more or less provided with material resources.

At the specific level of academic achievement, there is an improvement in transversal skills—especially digital literacy, but also communication and critical thinking—for the most motivated students, who are also those who show a greater propensity to participate during the lessons. Another part of the class, on the other hand, tends to “exile.” Non-participation is explicitly referred to material problems with devices and Internet connection: material difficulties that made it impossible for the teacher to intervene and which it is plausible to assume have weighed on the students' motivation and interest in actively participating during distance learning lessons.

Especially with reference to the more difficult students who could be defined passive-demotivated for behavioral traits and reaction to the situation, participation in DaD was defined as imperceptible and passive. Demotivation to learning, in some cases, may represent a consequence of performance anxiety disorders, linked to the health emergency in progress and to the relationship difficulties associated with it, with very strong consequences on the predisposition to resume school rhythms with serenity (in DDI) and to fit into the class group. Students' demotivation and performance were also greatly affected by students relationship with their parents, who were present but not motivating: for some, there was a tendency to interfere in matters of profit assessment, ordinarily the responsibility of the teachers; for others, there was a propensity to interfere, with suggestions, during questions or class assignments. Logistical difficulties—lack of space and the presence of other family members attending DaD or remote working—and Internet connection difficulties also negatively affected the pupils' motivation.

#### Depowered teaching

In the last configuration, DaD/DDI appears as a response dictated by the emergency: device problems (experienced by the students) and poor technological and infrastructural endowment of the school are factors that influence the course of the lessons (in terms of duration, possible thematic insights and desirable involvement of the students) and the expectations of teachers, students and their families. The delivery of training content is deficient in the way it is organized, due to infrastructural problems.

The organization of DDI was affected by a series of scheduling difficulties, partly related to the shortage of space (classrooms were not always capacious and therefore capable of guaranteeing spacing; LIMs (Interactive Whiteboards) did not always guarantee perfect interaction between in-presence and distance groups); partly due to the interruption of laboratory activities, given the re-functionalization of the spaces adopted to comply with the anti-COVID regulations (very often, laboratories and libraries were used to accommodate the largest classes). The lack of digital skills, especially in teachers with a higher average age, contributed to slowing down the school's process of adaptation to digital teaching: it could happen that entire lesson hours were skipped due to technical impediments (malfunctioning of the links for connecting to the virtual classrooms, obsolescence of the devices, drops in connection, etc.) that diverted the students' attention, inevitably reducing their motivation.

In general, there was a reduction/limitation of written skills and a prevalence of the assessment of oral skills, which can be explained by virtue of the multiple organizational difficulties of managing remote assessments (difficulties that, in part, would seem to be due to connection problems and in part would seem to be consequent to the non-transparent relationship of the students during the assessment of learning, such as attempts at defection and collaboration during tests). 2nd year classes were the ones that suffered more than all the other classes in terms of assessment, with a general decline in the level of learning. Also, in this case there was a change in the assessment criteria, with the introduction of an emphasis to the relational aspects linked to participation, dialogue and interaction between the students while carrying out the tasks.

For teachers, digital teaching has acted as a sounding board for pre-existing fragilities, amplifying the discomfort of the less fortunate, some of whom “got lost” because of the most diverse reasons: unjustified absences, performance anxiety, isolation. The organizational decisions taken during the DDI period, and the choice to alternate the weeks that some students attended online while other at school and *vice versa*, contributed even more to the breakdown of the class group.

These lost students (who could be defined opportunist-closed students) experienced a withdrawal into themselves. They lived in a comfort zone; they derogated from relationships. This closure is due in part to the absence of motivation to study and of family support, in part to fragile backgrounds with a lack of good Internet connection and of a suitable study environment.

The *extensive research*, carried out in the years 2022/2023, responds to the aim of reconstructing the main changes in the three dimensions of interest in the research (teaching practices; learning modalities; social relations) through a comparison between the pandemic emergency phase (background research) and the phase of exit from the emergency (extensive research).

Starting from the seven schools included in the previous research phase, a total of 14 schools were involved in this phase. The following were carried out in the schools: (i) One hundred eleven interviews with the teacher coordinators of the 10 selected classes; (ii) Thirty-one focus groups—for a total of 154 students involved. Interviews with the families of the students involved are currently in progress.

## Discussion

### Opportunities and criticalities of distance learning

In the Box 1, it's possible to analyze the types of teaching.

**Box 1:** Types of teaching.

Innovative teaching	DL/BL is an opportunity; teaching/educational practices are innovative; students perform highly
Traditional teaching	the DL/BL is an adaptive reaction; the (distance) setting up of the classic traditional lesson can have both positive effects on the more motivated students (and negative effects on the relational level). Horizontal interaction between students seems to be penalized, while the only possible interaction is vertical (between teacher and student)
Depowered teaching	the DL/BL is an emergency response; problems of devices, scarce technological and infrastructural resources, influence the course of lessons (in terms of duration, possible thematic insights, desirable student involvement…) and the expectations of teachers, students and families

The analysis of the first data obtained from the background research made it possible to highlight some opportunities and criticalities of the use of digital technologies in Distance Learning. In the teachers' experience, the fast interconnection to the new digital learning environments has had the merit of strengthening the relationship between schools and territorial realities (e.g., Universities and training organizations), widening and enriching the educational offer for students: “thanks to Google Meet we have allowed students to participate in conventions and conferences organized abroad… If it wasn't for the DaD experience we wouldn't have been able to do it!”. Interconnectivity and reduced distances to remote places had a positive impact on the range of students' activities: “DaD allowed us, for example, to connect easily with the United States.” Professional development is undoubtedly one of the most striking positive results of the last period; the intensive use of ICT has stimulated opportunities for dialogue and discussion among teachers on the most appropriate ways to respond to the needs of the “new school,” strengthening solidarity and cohesion within the school community.

The intensive use of ICT has led to an improvement in teachers' communication speed, collaboration and didactic innovation. The experimentation with digital technologies, albeit in an emergency phase, has enabled an unprecedented acceleration of the process of professional development and training, bridging the digital skills gap in teachers less inclined to the use of computer aids and devices in the classroom.

Together with the opportunities, a series of critical aspects referring to the period of emergency teaching can represent the keystone around which to reflect on the development of improvement interventions. These interventions invest the teacher's profession and strengthen the relationship of trust with the students, a true and proper load-bearing dimension for overcoming the difficulties encountered in times of pandemic. Quite apart from the difficulties of managing a distance lesson (due to the precariousness of the connection or to relational problems that made it complicated to affect the attention and concentration of the students—especially those who attended online in the DDI period), the production of audio-visual material can be a useful support to the lesson, provided that this commitment (in word) represents a deepening for the students and not the cause of an excessive extension of school time (a sore point, for the students, is represented by the afternoons spent listening to audio-recorded files; additional study hours added to the ordinary ones).

Among the criticalities attributed to the digital teaching experience, most of the interviewed teachers reported the negative impact of the loss of relational aspects on students' academic performance. In situations of emergency, of lack of alternatives, DaD/DDI would seem to have encouraged “the lazy to become lazy” (Gul_03). Attention and concentration deficits during lessons and loss on the personal decorum front (given the possibility of entering the classroom at the click of a mouse) are the consequence of that always-on condition, of perennial connectedness, which if experienced without awareness can subtract value from the educational and social dimension of school. On the social level, the laxity and withdrawal of the students into themselves, if on the one hand can be considered a consequence of the particular organization that digital education has assumed over the months, on the other hand can also be a reflection of social fragilities pre-existing in the pandemic period. The students were disoriented by the sudden changes (both managerial and organizational) adopted during the months in which DaD and DDI alternated. The students' laxity/refocus on themselves was compounded by learning problems due to poor attention and concentration.

## Concluding remarks

Within the innovative framework of the positive thinking evaluation (Lo Presti, 2020), the background research illustrated has made it possible to interpret the criticalities linked to the DaD experience as a possibility for a participatory reflection—with teachers and students—on the importance of innovating the return to school presence, enriching the educational offer with new instances and awarenesses that have emerged in a time of strong changes, still in the consolidation phase.

In a small way, the three different didactic configurations reproduce the enabling factors, otherwise hindering, that facilitated/depowered learning, its assessment and the emotional component linked to the specificity of interactions in the new digital learning environments. In spite of the difficulties in managing digital didactics, whether for technical reasons, motivation reasons, or linked to the cultural and family background of the students, the interviewed teachers agree in attributing to DaD the function of engine and driving agent of change in the school system. The intensive use of ICT and the guarantee of accessibility for all—teachers, students and their families—have undoubtedly enriched education in a variety of ways and with a variety of opportunities.

In conclusion, ICTs, employed in the age of digital didactics, can take on a propulsive function for change in schools because they have enabled/will enable an impact on the school setting and by transforming the learning environment through the enhancement of technological equipment (PC + LIM + camera in classrooms).

The digital didactic has redesigned space and reorganized time through the decompression of lesson duration, exploiting the opportunity to combine synchronous and a-synchronous even in the presence, using digital environments to enrich the frontal lesson. Digital technologies have improved, and will continue to improve, the processes of communication and socialization by stimulating, in students, the development of key competencies of digital literacy and media education and by strengthening (or laying the foundations for strengthening) communication with families; iii. have facilitated, and are expected to continue to facilitate, the work of correcting texts and assignments by teachers, making dialogue and reflection with students more interactive.

However, the initial results reconstructed represent only the start of an informed reflection on the trajectories of change that will be consolidated in schools in the coming years, and that much will depend on the evolution of the pandemic and the organizational decisions taken by ministerial bodies and individual educational institutions. The complexity of digital educational environments, which emerged from the research, draws attention to the importance of resorting to a reasoned, and not emergency, school planning, in which reflection on the use of digital devices, from being ancillary, becomes central within the educational curricula, in response to the increasingly felt need to consolidate the digitalization of educational processes, through a conscious use of digital tools and collaborative learning methods.

## Data availability statement

Raw data supporting the conclusions of this article will be made available by the authors, without undue reservation.

## Ethics statement

The studies involving human participants were reviewed and approved by Settore Privacy—Area Affari Legali Sapienza Università di Roma. Written informed consent to participate in this study was provided by the participants' legal guardian/next of kin.

## Author contributions

The author confirms being the sole contributor of this work and has approved it for publication.
